# A computational model to design neural interfaces for lower-limb sensory neuroprostheses

**DOI:** 10.1186/s12984-020-00657-7

**Published:** 2020-02-19

**Authors:** Marek Zelechowski, Giacomo Valle, Stanisa Raspopovic

**Affiliations:** 1 Center for medical Image Analysis & Navigation, Department of Biomedical Engineering, University of Basel, Basel, Switzerland; 2 Neuroengineering Lab, Department of Health Sciences and Technology, Institute for Robotics and Intelligent Systems, ETH, Zürich, Switzerland

**Keywords:** Sensory, Neuroprosthesis, Lower limb, Hybrid computational model, Neural interfacing, Neural stimulation

## Abstract

**Background:**

Leg amputees suffer the lack of sensory feedback from a prosthesis, which is connected to their low confidence during walking, falls and low mobility. Electrical peripheral nerve stimulation (ePNS) of upper-limb amputee’s residual nerves has shown the ability to restore the sensations from the missing limb via intraneural (TIME) and epineural (FINE) neural interfaces. Physiologically plausible stimulation protocols targeting lower limb sciatic nerve hold promise to induce sensory feedback restoration that should facilitate close-to-natural sensorimotor integration and therefore walking corrections. The sciatic nerve, innervating the foot and lower leg, has very different dimensions in respect to upper-limb nerves. Therefore, there is a need to develop a computational model of its behavior in response to the ePNS.

**Methods:**

We employed a hybrid FEM-NEURON model framework for the development of anatomically correct sciatic nerve model. Based on histological images of two distinct sciatic nerve cross-sections, we reconstructed accurate FEM models for testing neural interfaces. Two different electrode types (based on TIME and FINE) with multiple active sites configurations were tested and evaluated for efficiency (selective recruitment of fascicles). We also investigated different policies of stimulation (monopolar and bipolar), as well as the optimal number of implants. Additionally, we optimized the existing simulation framework significantly reducing the computational load.

**Results:**

The main findings achieved through our modelling study include electrode manufacturing and surgical placement indications, together with beneficial stimulation policy of use. It results that TIME electrodes with 20 active sites are optimal for lower limb and the same number has been obtained for FINE electrodes. To interface the huge sciatic nerve, model indicates that 3 TIMEs is the optimal number of surgically implanted electrodes. Through the bipolar policy of stimulation, all studied configurations were gaining in the efficiency. Also, an indication for the optimized computation is given, which decreased the computation time by 80%.

**Conclusions:**

This computational model suggests the optimal interfaces to use in human subjects with lower limb amputation, their surgical placement and beneficial bipolar policy of stimulation. It will potentially enable the clinical translation of the sensory neuroprosthetics towards the lower limb applications.

## Background

Leg amputees lack sensory feedback and have limited voluntary control of currently available prostheses [1]. These limitations do not allow for a correct generation of postural reflexes at the spinal level and overall correct sensory-motor integration between the user’s central nervous system and the artificial limbs. Because of the lack of sensory feedback and no controllability of the prosthesis itself, which are difficult to separate as issues, since inherently connected, amputees are suffering many health-related problems. Users experience dangerous falls [2], do not manage to maintain symmetry during standing and walking [3, 4], i.e. they tend to shift more weight and have a prolonged stance phase on the sound limb than on the prosthetic limb [5–7]. Resulting abnormal kinematics and postural asymmetries can, after long-term use of the prosthesis, lead to musculoskeletal diseases as knee and hip osteoarthritis, osteoporosis, and back pain [8, 9]. Moreover, since they exert unnatural compensatory movements with prosthetic and healthy leg and body, they face an augmented metabolic cost, then fatigue and occasionally hearth failures [10]. As such, an amputee, especially a thigh-level one (transfemoral (TF)), is faced with several challenges in daily life situations. Sitting and standing up, running, shuffling and carrying loads can be a difficult and even dangerous task for a TF amputee. Moreover, 50–80% of amputees report neuropathic pain from the missing extremity, which is called a phantom limb pain (PLP) [11] and for which an effective treatment is not available [12]. Finally, the users do not perceive the prosthesis as part of their own body, which increases the cognitive effort when using the device itself [13], affecting its acceptability (low embodiment) [14, 15] and causing a reduction in the confidence of the subject in its use (i.e. they are afraid to fall if relying over it) resulting in 60% of lower limb amputees abandoning the prosthesis (i.e. they do not use it and do not walk anymore) [16, 17]. Sensory feedback provided by foot sole mechanoreceptors is important for controlling balance and movement in humans [18–22]. Lower-limb amputees rely on often-uncomfortable haptic feedback from the stump-socket interaction to monitor ground contact, counteract interaction with obstacles, stabilize balance and walk symmetrically. Many, of the drawbacks associated with operating the device arise from the lack of proper sensory feedback of the lost limb. Partial or full restoration of the afferent information path would allow closing that gap, which currently stands wide open. Recently, the provision of sensory feedback, has been shown to alleviate the PLP and metabolic cost in transfemoral amputees while walking [23], and help regarding the fall avoidance, stair mobility and embodiment boosting [24]. These are important rationales for the development of the models for a sensory neuroprosthesis, as the present one.

Sensations can be restored by means of non-invasive techniques such as electrotactile [25] and vibrotactile [26] stimulations, with the drawback of being not homologous and not selective, and therefore of increasing the cognitive effort of the subjects and forcing them to spend a period of training to only partially overcome this limitation. By connecting to the peripheral nervous system with a neural interface [27], it is possible to restore close-to-natural sensations within bidirectional loop as recently showed in upper-limb amputees [28–30].

Recently, very important clinical translations have been shown in the upper-limb amputees’ investigations [28–38]. Neuromodulation at the median and ulnar nerves using transversal intraneural electrodes (TIMEs) [28, 31–34], allowed amputees to feel touch sensations from a missing hand and to exploit this sensation in prosthesis bidirectional control [28, 32, 35], diminished their phantom limb pain [32] and boosted prosthesis embodiment [29, 32, 36, 37, 39]. A long-term use of FINE electrodes in humans has been reported [29, 30, 38, 39]. Despite these achievements, the sensations encoding mechanisms, the most effective way to restore sensory feedback by invasive neural stimulation, are still objects of a scientific discussion [33, 34, 40, 41].

Here we explored how these technologies, namely TIMEs and FINEs, could be transferred to the lower limb application, via computational modeling. The development of an optimal communication between neural (ions) and artificial (electrons) codes (i.e. electrode-nervous tissue communication), based on deep understanding of Electro-Neuron interactions is needed. It is a mandatory step, since the dimensions of median and ulnar nerves (upper limb) are much smaller than the ones of the sciatic nerve. Existing models of nerves (that do not include human sciatic nerve for sensory stimulation) are exploring the effects of the nerve stimulation to the resulting neural population [31, 42, 43]. What is missing is a sensory nerve model, which would indicate how to optimally stimulate within the high-dimensional space of possible electrode’s geometries, stimulation parameters and their placements within PNS, intractable with the “brute-force” approach. To address this, we developed a detailed anatomically and biophysically plausible model of the human sciatic nerve, accounting both for the electrical stimulation effects and the neural responses of axons: electro-neuro model (ENM). We compared the TIME and FINE electrodes [27] in terms of efficiency (selectivity) and efficacy (the threshold values).

This model was used to identify i) the optimal geometry of the neural interface, ii) neurosurgical placement (number of implants) and iii) beneficial stimulation policy. The type of electrode, number of active sites (AS), the number of devices to be implanted and more sophisticated stimulation policy, are explored in the present study.

## Methods

**Fig. 1:**
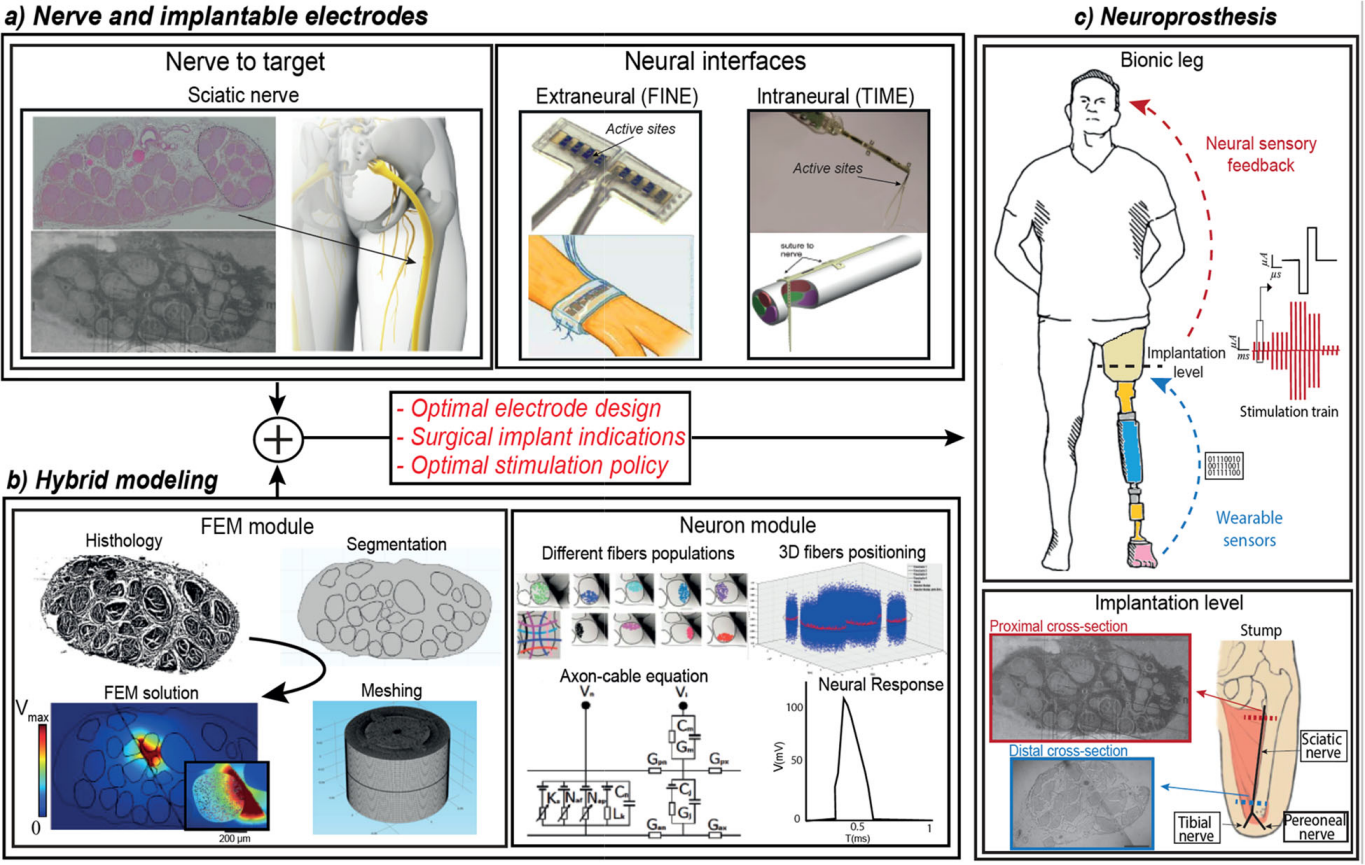
Schematic representation of hybrid modeling for neuroprosthetic applications. **a** The target peripheral nerve is identified for restoring sensory feedback (i.e. sciatic nerve) and its histological pictures are extracted. In order to interface the neuroprosthesis with the biological tissue, intraneural and extraneural interfaces are considered (i.e. FINE and TIME). **b** Hybrid models are developed considering geometrical and physical properties of nerve-electrode interface during neural stimulation (FEM module). Axon fibers model and different populations are integrated to study recruitment and electrode selectivity (Neuron module). **c** The outcomes of this process, guiding design of an optimal neuroprosthetic leg for trans-femoral amputees, are: Optimization of the electrode design; Indications for the surgical implant; and optimization of the stimulation strategy

We developed ENM of sciatic nerve that will allow for evaluation of different electrode designs and operating paradigms (Fig. 1).

### Finite elements model (FEM)-neuron hybrid model

In our study, we utilized a three-step framework [44, 45], combining a realistic Finite Elements Model (FEM) of the nerve, providing electric potentials, with a Neuron Compartmental Model for calculation of fiber recruitment. The experimental setup was similar to the one described previously [46] and optimized for computational time.

### Electrical potentials solution using FEM

To accurately replicate the anatomical structures of a human sciatic nerve, two histological cross-section images were identified for the model creation (Fig. 1.). The first image [47] representing the nerve at the ischial tuberosity, later referred to as the proximal anatomy, and another one close to the sciatic bifurcation [48] – denominated as the distal anatomy. The proximal geometry (187 × 88 mm) of a 28-year-old patient contained 37 fascicles and should be an attainable spot for electrode placement for even the highest trans-femoral amputees. The distal cross section of a female cadaver (87–102 years old) accommodated 31 fascicles at 58.2 mm^2^ (11.5 × 6.4 mm). This level of the nerve is suitable implantation sport for the lower above-knee amputations.

Images were imported into ImageJ software [49] for manual segmentation of the anatomical structures with a NeuronJ plugin [50]. Exported data contained the outline of the nerve and the fascicles within it. Next, we reconstructed the anatomical features of the nerve using MATLAB (The MathWorks, Inc., Natick, Massachusetts, United States). The outer layer of the fascicles – perineurium, was defined as 3% of its diameter [51], and the endoneurium filling the rest of the fascicle’s lumen. The segmented 2D geometry was then imported into COMSOL Multiphysics (COMSOL AB, Stockholm, Sweden) FEM software, in which by extrusion in the longitudinal direction, a 3D model was created.

We prepared a total of 15 different electrode designs (7 extraneural and 8 intraneural) with varying number of active sites and dimensions.

Intraneural models were based on the Transversal Intrafascicular Multichannel Electrode (TIME) [52], and we built 4 active sites configuration for each nerve model – 12, 16, 20 and 24 ASs, spanning across the length of the electrode’s shaft. Following the Raspopovic et al., 2017 [46] we have opted for the asymmetrical design of the electrode with the ASs on the opposite side shifted by a half the distance between the sites. Using this approach, we maximize the effective spatial range of the electrode, with respect to the symmetrical case. We adjusted the size of our models to fit the anatomical structures of the sciatic nerve. Therefore, the intraneural electrodes were 18 and 10 mm for proximal and distal anatomy respectively. The thickness was fixed at 20 μm for all the designs, while the width depended on the number of active sites to account for the needed trace paths to the stimulation point (380 to 670 μm range). The AS area was modelled as a circle and has a 60 μm diameter [52].

The extraneural electrode’s design was based on the no-compression version of Flat Interface Nerve Electrode’s model (FINE) [43]. Size of the electrode was adjusted to match the dimensions of the two nerves - 18.75 × 8.85 mm for the proximal and 10.9 × 7 mm for the distal anatomy. Since neither the compression model of the nerve, nor histological data of human sciatic nerve under compression were available, we opted to implement the no-compression version of FINE (a nerve and FINE have the same dimension). Yet, implemented model is clinically relevant, since being qualitatively similar to the extraneural electrodes, which are not compressing the nerve, used in the unique effort performed to interface sciatic nerve for sensory feedback, until today [53]*.* The contact area of active sites was modelled as in the original FINE design – 0.5 × 0.5 mm. Our extraneural electrodes had 12, 16, 20 and 24 active sites for the proximal anatomy of the nerve and 12, 16 and 20 ASs for distal as we were limited by the dimensions of the nerve (maintaining the original AS’s sizes).

To correctly calculate the electric potential distribution within the model, we needed to attribute each tissue with a corresponding electrical property [54]. Epineurium was defined as an isotropic medium with a conductivity value (σ) of 0.0826 S/m [42, 55]. Intrafascicular endoneurium is assumed as an anisotropic tissue with a conductivity tensor of 0.571 S/m and 0.0826 S/m [42, 55], for the longitudinal and transversal values respectively. Perineurium’s value was set to 0.00088 S/m as reported in Raspopovic et al., 2017 [46]. As reported in previous studies [42, 43, 55], the space closely surrounding the nerve was modelled as a homogenous saline solution with a conductivity of 2 S/m. Main shaft of the electrode was defined as a polyimide structure with σ = 6.67*e-14 S/m [52]. The boundary current conditions were replicated from the previous study [46] – cylinder with a 16 mm diameter and 15.4 mm length in both directions from the center. The active sites of each electrode were defined as a boundary current source with an effective current of 2 μA for TIME and 20 μA for our extraneural electrode (FINE). Thanks to the linearity of governing equations results for the other values of current can be simply linearly scaled.

The nerves and the electrode models were then merged in the COMSOL software, and using the EC module of COMSOL, an equation to the electromagnetic problem was defined as a Laplace formulation for the extracellular electric potential:

$$ \nabla \ast \upsigma \nabla {\mathrm{V}}_{\mathrm{e}}=0. $$

The solution is discretized based on a mesh generated for the model [56]. To reduce the computational complexity, the mesh composed of tetrahedral elements with an extremely fine density in the proximity of the electrode (higher electric field gradient) and coarser for the rest of geometry is implemented. To automatize the process of running FEM simulations we utilize the COMSOL interface available for MATLAB – COMSOL Link with MATLAB.

### Axonal responses calculation via NEURON model

In our study, we utilized the compartmental neuron model with Ranvier nodes and axon tracts separating them. In particular, we use a McIntyre-Richardson-Grill model [57]. Each fiber of diameter (D) consists of 21 nodes of Ranvier (shifted randomly across fiber population) and 20 internodes with a distance of L = 100 ∗ D between them. The NEURON’s extracellular stimulation procedure was used to simulate the excitation of the cells.

To account for the anatomical variability of the sciatic nerve, we implemented multiple fiber populations per fascicle, similarly as in [46], since fibers within one fascicle may account for sensation from different areas of the leg, and/or can be very concentrated or uniformly spread over the fascicle. Depending on the size of the fascicles, 1, 3 or 5 populations were placed in the fascicle’s lumen (small < 400 μm, 400 μm < medium < 800 μm, big > 800 μm). Each population occupied a different area of the nerve bundle, but its fibers remained grouped. The density and the diameter distribution of the fibers were taken from Garven et al. [58] and match a 28-year-old female patient. Fiber density was reduced from 11,953 to 240 fiber per mm^2^ (a 50x reduction), similarly as in other works [43, 46], which vastly improved our simulation times. This reduction does not affect potential distribution within the individual fascicle. Importantly, we maintained the fiber diameter distribution, therefore accurately representing nerve’s overall functional anatomy and neural responses.

### Connecting FEM and NEURON into a hybrid model

Solution to the electric potential distribution calculated for the FEM structure was interpolated to the desired positions of fiber nodes of Ranvier, as explained in detail [46] and exported through COMSOL Link with MATLAB for further steps. Interpolated data points were then sent individually for each fiber within a given fixed range away for the active site. The neuron’s response is later computed using NEURON’s MRG model and the extracellular mechanism for membrane depolarization [59]. We iterated 60 times for each fiber, gradually increasing the amplitude of the electric potential at the Ranvier node, maintaining the 50 μs pulse duration, effectively changing applied charge from 0.5 to 60 nC. Axon was considered recruited, when a generated action potential run over the entire length of the neuron.

### Varying operating modes (policy of stimulation) and multi-electrode implantation

As neural interfaces allow stimulating through more than one active site at the same time, we evaluated different stimulation protocols. We tested single active sites for a monopolar cathodic stimulation (which is conventionally used in almost all neuroprostheses) and then used a superposition to evaluate bipolar modes (see Fig. 6a). Highlighted areas schematically represent potential distributions (A, B, C) elicited by different active sites, which are disposed as explained in continuation. A indicates a field potential elicited by a single AS. B indicates a field potential elicited by adjacent ASs, which is on the opposite side of the electrode with respect to A. C is elicited by the AS closest to the A on the same face of the electrode. In each configuration, we simulated different polarities of the individual ASs, switching between positive (indicated by the red color) and negative (blue color), effectively changing the potential distribution field in the nerve. Monopolar stimulation allows for an activation of only one active site at a time, while bipolar stimulation enable to use two contacts in any polarization configuration (see Fig. 6a: e.g. opposite colors red and blue, and same polarization–color red). As an example, enabling bipolar stimulation allows applying opposite current to the adjoining active site and therefore modified the spread of the current (Fig. 6a right inset).

Additionally, we investigated the effects of implanting multiple intraneural electrodes on the overall fascicle recruitment. This may serve as an indicator for surgeons to choose the best approach for a given target anatomy. For both the proximal and distal anatomy we simulated an insertion of up to 4 electrodes.

### Performance evaluation

Each electrode’s variant and the operating protocol was assessed based on the two performance indexes to select the most optimal approach for neural stimulation. The aim was to design the most effective neural interface to selectively recruit fascicles within a given anatomy. Therefore, to define the selective recruitment we used two separate indexes that considered both the percentage of fascicle’s recruitment as well as the absolute number of fibers recruited. The first index [42] evaluates the spatial selectivity and measures if the fascicle i is selectively recruited with respect the entire fascicles range:

$$ {\mathrm{Sel}}_{\mathrm{i}}={\upmu}_{\mathrm{i}}-\frac{1}{\mathrm{m}-1}\sum \limits_{\mathrm{j}=1,\mathrm{j}\ne \mathrm{i}}^{\mathrm{m}}{\upmu}_{\mathrm{j}} $$

where μ_i_ is the number of axons recruited employing extracellular potential divided by the total number of fibers within the i^th^ fascicle.

Based on the principle reported in Van Hees and Gybels 1972 [60], that even a single activated fiber can elicit a sensation (a “tactile unit”), we use an additional selectivity index proposed in Raspopovic et al., 2017 [46]. It aims to evaluate the functional, sensory, selectivity of an active site:

$$ \mathrm{Sel}\_{\mathrm{s}}_{\mathrm{i}}=\frac{{\mathrm{n}}_{\mathrm{i}}}{\sum_{\mathrm{j}=1}^{\mathrm{m}}{\mathrm{n}}_{\mathrm{j}}} $$

where n_i_ is the number of activated fibers within the i^th^ fascicle, while n_j_ is the total number of elicited fibers. Both indexes are calculated for each active site and for each fascicle. AS was considered selective when it respected both spatial and functional selectivity condition (Sel_i_ > 0.6 and Sel _ s_i_ > 0.9) and then added to the electrodes score.

When we performed the validation process, we assumed the threshold to be a charge value at which 10% of axons within the fascicle are recruited [61].

### Computational optimization

The entire population of fibers in the proximal nerve reached 13.5 K in 37 fascicles, which is a significant number to compute for each simulation (12–24 simulations per single electrode). Considering our stimulation parameters, we created a test setup to estimate a maximal effective range of stimulation (range between eliciting a single fiber and an entire fascicle without activating the others), and avoided simulating out from it, since it would waste the computational time, while being useless for the selective stimulation. Out from this range, the fiber would either not have been recruited, or it would not be possible to elicit a fascicle-selective stimuli (sensation), effectively discarding it from the selectivity consideration. The setup assumed performing multiple simulations of the entire nerve’s population and evaluating the fiber recruitment. We have decided that eliciting a single fiber within the fixed range, would implicate an entire fascicle, it belongs to, for the selectivity consideration.

### Statistical analysis and system specification

All data were extracted and processed in MATLAB. All statistics were performed using available built-in functions. The normality of the data was first checked (one-sample Kolmogorov-Smirnov test) and reported the average and standard deviation. Since none of the data was normally distributed, for the analyses in the paper a two-tailed Kruskal-Wallis test was used to measure the significance of the chi-square statistic. When needed, a Tukey’s Honestly Significant Difference Procedure for multi-group comparison was applied. All the software simulations were ran using on a mid-range PC (HP Z2, Intel i7–8700, 32GB RAM, Windows 10). The software used included MathWorks MATLAB 2017b, COMSOL Multiphysics 5.4, NEURON v7.3 and ImageJ v1.48.

## Results

An intraneural electrode (TIME, [62]) and an extraneural electrode (FINE, [63]) were selected since they were used in many clinical investigations in upper-limb [28, 29, 31, 32, 38, 53]. The abovementioned electrodes are implanted and simulated into two different parts of the sciatic nerve: proximal and distal section (see Methods section). This choice was taken in order to consider the different levels of amputation that could occur in trans-femoral amputees and also to exploit our model for two different nerve geometries.

**Fig. 2:**
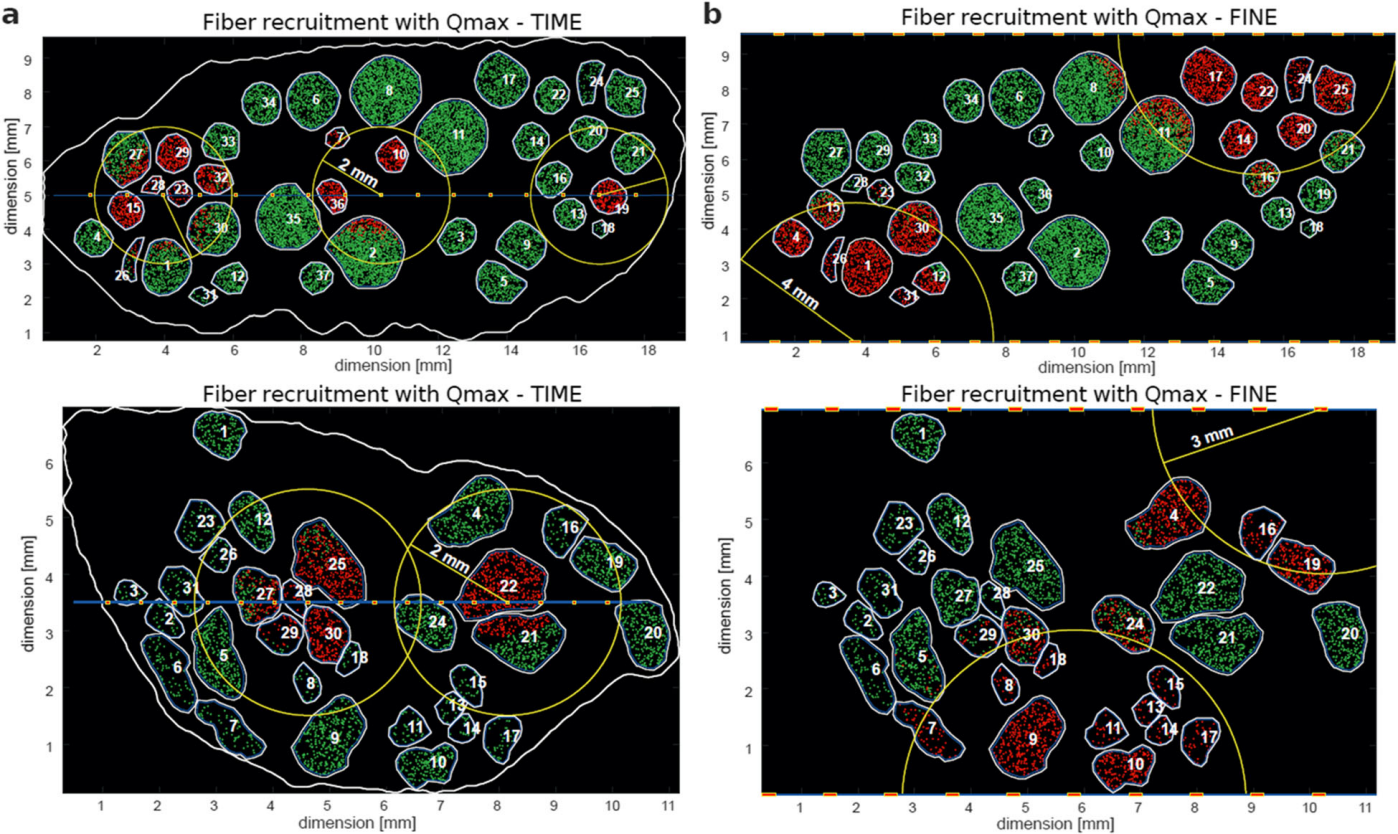
Optimization of the simulations. **a** Maximal range of stimulation (yellow circle - 2 mm) for TIME electrode in distal and proximal cross-section of the sciatic nerve. **b** Maximal range of stimulation (yellow circle - 4 mm for proximal and 3 mm for distal sections) for FINE in distal and proximal cross-section of the sciatic nerve. Red dots indicate recruited fibers at maximum charge (Qmax). Green fibers are not recruited. If a fiber is contained inside the range, entire fascicle is taken into account

First, in order to optimize the computational burden, we tested an optimal range of distances from the active site for both geometries - proximal and distal, as well as for an electrode type – intraneural and extraneural. After running 32 (proximal) and 20 (distal) simulations for the extraneural designs, and 32 (16 for each proximal and distal) using intraneural, for entire nerve’s fiber population, we evaluated results in terms of fiber activation and significance to the selectivity calculations. In both cases for TIME variants, we have observed no meaningful fibers recruited above 2000 μm away from the active site (see Fig. 2a).

With extraneural designs we noted a discrepancy between the proximal and the distal anatomy, being a consequence of a size difference among them (see Fig. 2b). Therefore, we assigned an effective range of 4000 μm for the proximal and 3000 μm in case of the distal anatomy. Table 1 shows the absolute fiber count reduction per single electrode simulation (TIME20 in the proximal nerve) and the time improvement we gained over the previous approach.

**Table 1:** Computational time difference shown for TIME20 intraneural electrode in the proximal nerve simulations

proximal TIME20	standard setup	range optimized	Reduction in computational time
**fiber count**	271,280	53,132	**80.41%**
**computational time [h]**	226.1	44.3	

**Fig. 3:**
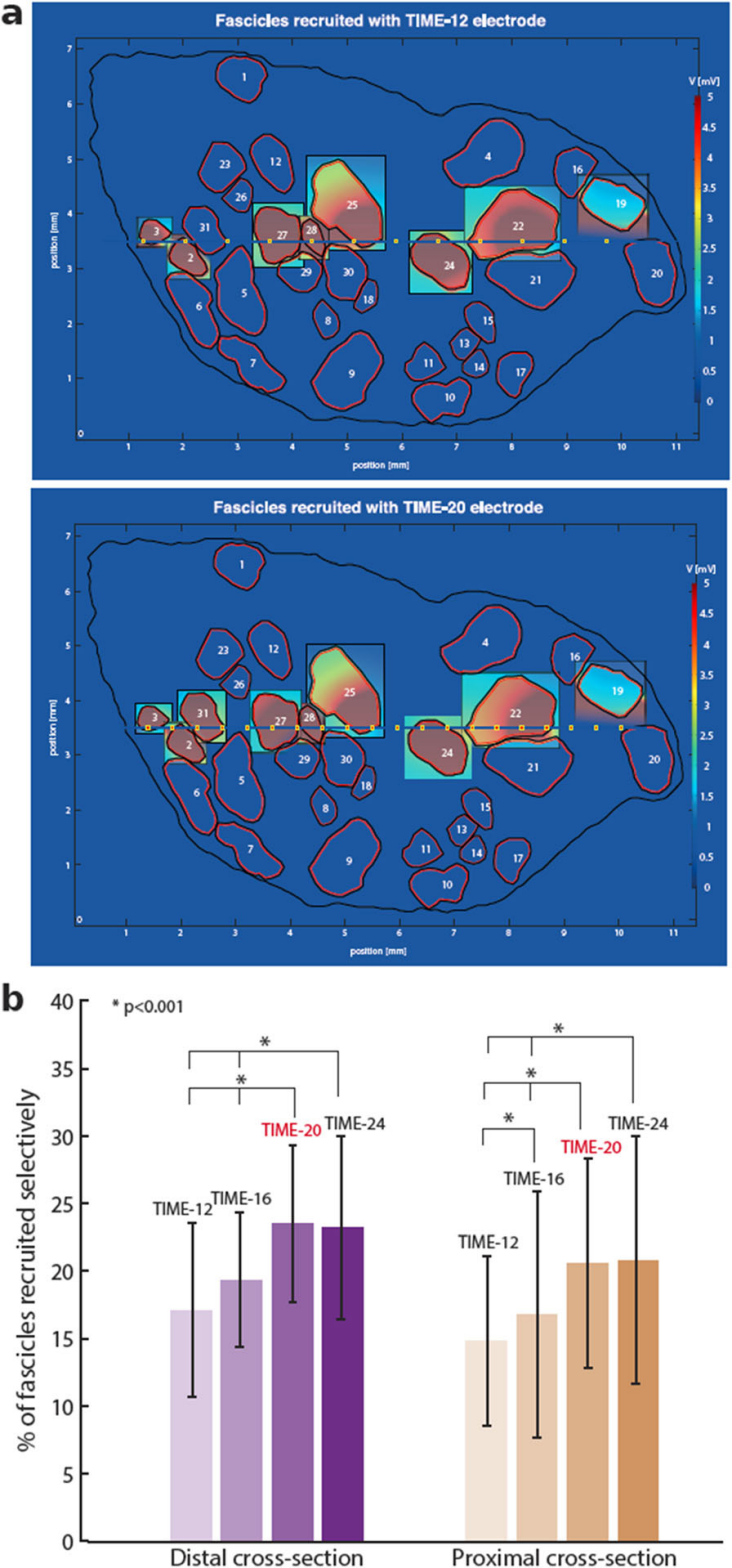
Optimal number of active sites for TIME. TIME models with 12 and 20 ASs are represented in panel (**a**). Highlighted insets represent the fascicles selectively stimulated. The elicited voltage distributions are plotted in the planes orthogonal to the center of stimulating AS. Two sciatic nerve’s anatomies were used to create hybrid models for the estimation of electrode’s performance proximal and distal cross-section. **b**) A bar graph presents percentage recruitment of fascicles for TIME with varying number of active sites, from 12 up to 24. The increase from 16 to 20 ASs yields a significant augmentation in the number of selectively stimulated fascicles (* *p* < 0.001), while when passing from 20 to 24 ASs there was no statistical difference (*p* > 0.05), for both anatomies

With this optimized model, we first investigated the optimal number of active sites for TIME (Fig. 3). FEM solutions for distal anatomy of the sciatic nerve with 31 fascicles are shown (Fig. 3a). The fascicles selectively stimulated were calculated and their percentage in respect to the total number of fascicles were obtained for TIME with 12, 16, 20, and 24 active sites for both nerve’s cross-sections (Fig. 3b). The smallest number of targets was reached using TIME with only 12 active sites (6 on each side of the electrode) – 19.35 ± 4.9% for distal and 14.86 ± 6.3% for the proximal section.

For the distal section, increasing the number of active sites did not result in more fascicles recruited passing from 12 to 16 AS (Kruskal-Wallis test with Tukey-Kramer post-hoc test, *p* > 0.05), but number of fascicles increased– 23.55 ± 5.8% (*p* < 0.001), when using 20 AS. The effectiveness did not change significantly with TIME-24, becoming 23.22 ± 6.8% (p > 0.05). Therefore, the configuration with 20 AS, being more effective than smaller number of AS and equally effective as higher number of AS is chosen as an optimal one.

Cross-section of the proximal anatomy consisted of 37 fascicles and the same electrode configurations were tested (Fig. 3b right side). An increase in effectiveness was observed for TIME with 16 AS, where 16.76 ± 9.1% nerve was successfully targeted (p < 0.001). TIME with 20 stimulating sites showed higher performance among previous variations with the number of selectively activated fascicles of 20.54 ± 7.7% of all fascicles. The effectiveness did not vary significantly for the proximal anatomy when passing to the TIME-24 to 20.81 ± 9.2% (*p* > 0.05). The results demonstrated that the optimal number of active sites, in terms of stimulation selectivity, for TIME in sciatic nerve is 20 (10 per side).

**Fig. 4:**
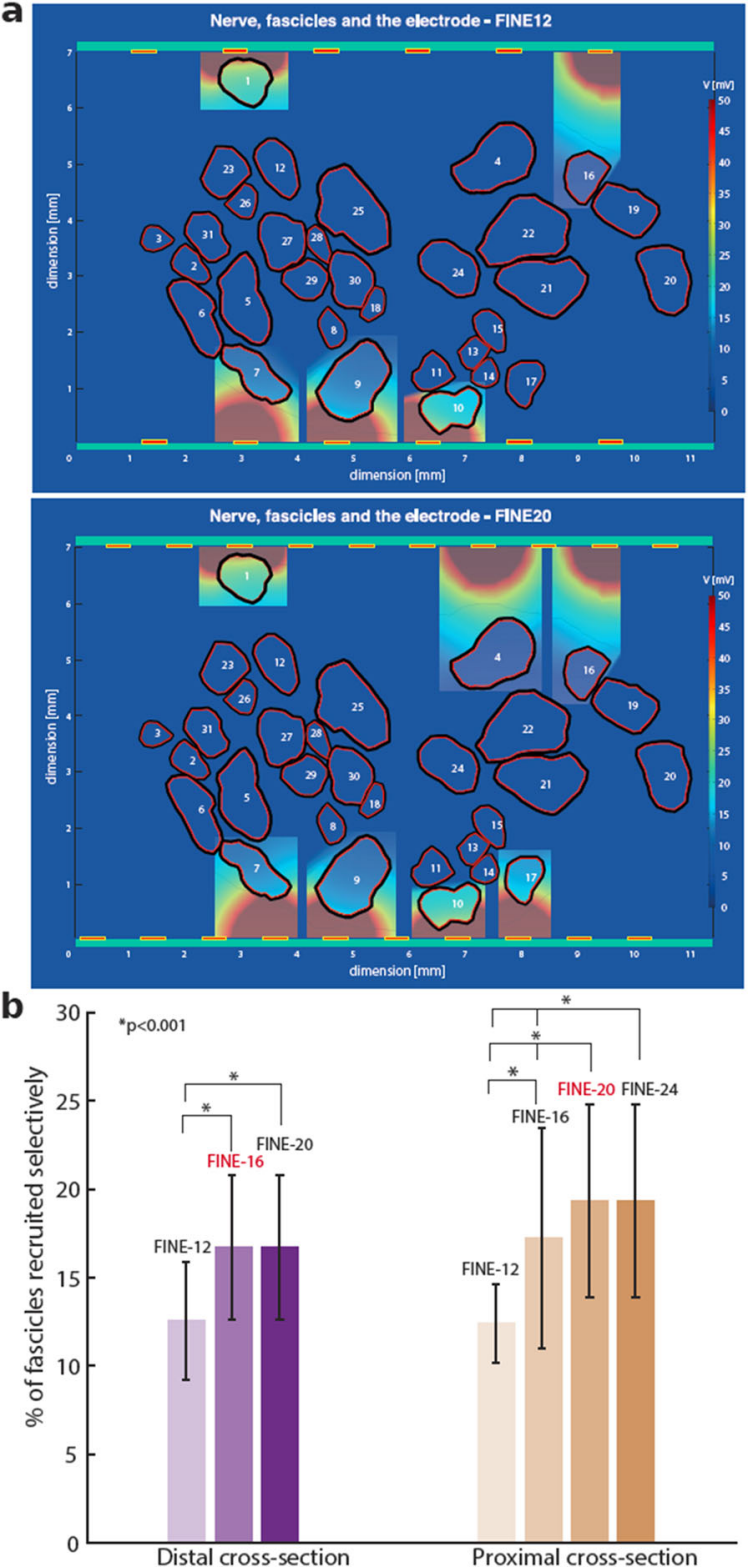
Optimal number of active sites for FINE. FINE models with 12 and 20 ASs are represented in panel (**a**). Highlighted insets represent the fascicles selectively stimulated. The elicited voltage distributions are plotted in the planes orthogonal to the center of stimulating AS. **b**) A bar graph presents percentage recruitment of fascicles for FINE with varying number of active sites, from 12 up to 24. The increases from 12 to 16 AS for distal and from 12 to 20 AS for proximal section yields significant increase in the number of selectively stimulated fascicles (* *p* < 0.001)

Similarly as done with TIMEs, a FINE was simulated for both sciatic nerve sections (Fig. 4). For both anatomies FINE with 12 active sites presented the worst performance, with only 12.43 ± 2.3% and 12.58 ± 3.4% for proximal and distal anatomy respectively. Additional 4 active sites gave a significant selectivity improvement: 17.29 ± 6.3% for the proximal and 16.77 ± 4.1% for the distal anatomy were reached selectively. While further increase in number of active sites, to FINE-20, for distal anatomy did not change significantly the final number of fascicles recruited, 16.77 ± 4.1%. Instead, for proximal cross-section FINE-20 boosted the success rate to 19.46 ± 5.5%. In the proximal section, for FINE-24 no improvement was observed respect to FINE-20 – 19.46 ± 5.5%. The results demonstrated that the optimal number of active sites, in terms of stimulation selectivity, for FINE in the distal part of the sciatic nerve is 16 and 20 for the proximal.

**Fig. 5:**
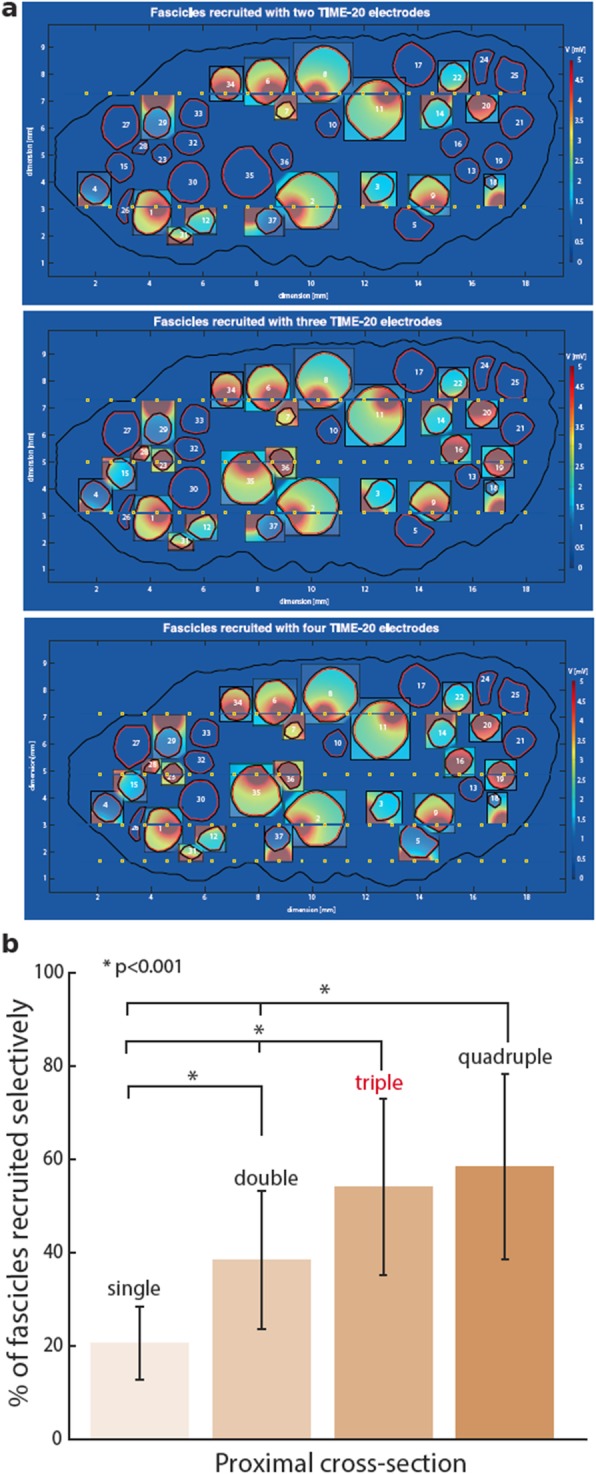
Optimal number of implants. **a** TIME models with double, triple and quadruple implants are represented in panel (**a**). Highlighted insets represent the fascicles selectively stimulated. The elicited voltage distributions are plotted in the planes orthogonal to the center of stimulating AS. **b** A bar graph presents percentage recruitment of fascicles with varying number of implants, from 1 up to 4. The increase from 2 to 3 implants yields a significant augmentation in the number of selectively stimulated fascicles (* *p* < 0.001)

After the optimization of the neural interface, we investigated the number of intraneural electrodes to implant in order to selectively stimulate as many fascicles as possible inside the nerve, consequently maximizing the efficacy of the neural stimulation (i.e. increase the probability to elicit several distinct sensation locations referred on the phantom leg). On the other hand, interfascicular electrodes are quite invasive and therefore implanting too many electrodes may cause unnecessary nerve damage. To unveil this effects, single, double, triple and quadruple TIME implants were simulated and compared in the most challenging case of very high amputations, and therefore in the proximal section (Fig. 5). Since 20 AS was found as the optimal number of active sites, TIME-20 was inside the sciatic nerve and the number of fascicles selectively recruited was evaluated.

The results for implantation of multiple TIMEs is shown in Fig. 5a, b. We observed big leap from single to two electrodes implanted in parallel: 20.54 ± 7.7% of fascicles selectively recruited for single and 38.38 ± 14.7% for double implant (*p* < 0.001). Placing the third electrode is beneficial for the selectivity improving the percentage of fascicles recruited to 54.05 ± 18.9% (*p* < 0.05). Implanting a fourth TIME was not effective, since the performance did not change significantly – 58.37 ± 19.8% (*p* > 0.05).

Taking into consideration these results together with the potential nerve damage and the complexity of surgical procedure, it is not beneficial to implant more than three TIME in a human sciatic nerve.

**Fig. 6:**
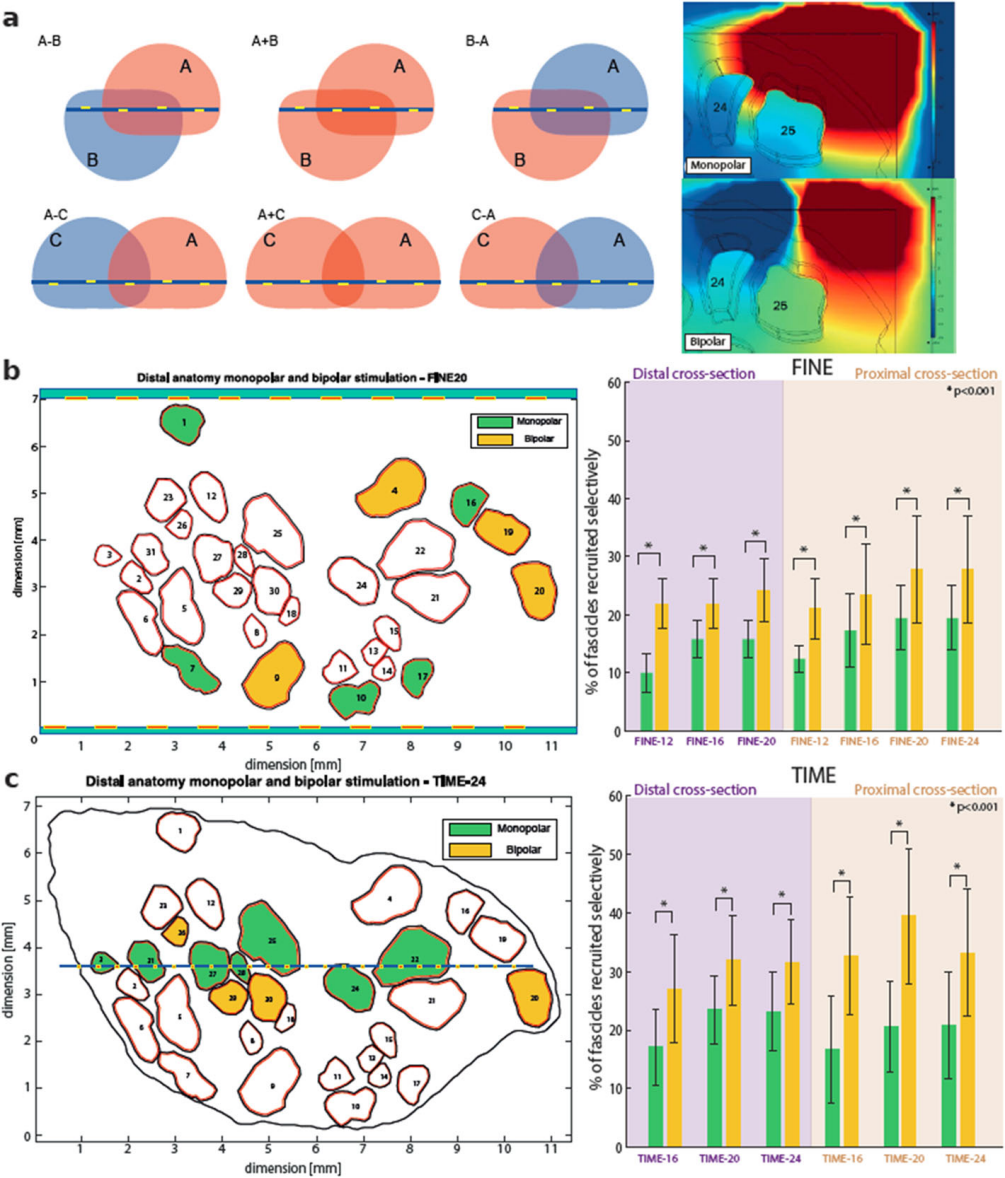
Optimization of stimulation strategy – Bipolar VS Monopolar stimulation. **a** Different bipolar stimulation configurations are schematically displayed (left). Red and blue areas represent schematically voltage distribution induced by a given AS (red for positive and blue for negative polarity). A indicates a distribution elicited by a single AS. B indicates a distribution elicited by adjacent ASs, which is on the opposite side of the electrode with respect to A. C is elicited by the AS closest to the A on the same face of the electrode. Voltage distribution elicited by an active site using monopolar stimulation and two adjacent sites using bipolar stimulation (case of A-B) are computed (right). **b** New fascicles are selectively elicited by bipolar stimulation (in yellow) with respect to monopolar (green), for both TIME and (**c**) FINE. **b-c** Bar plots of FINE and TIME with different number of actives site are shown, in which the bipolar has always a higher selectivity than the monopolar stimulation

Then, we investigated the optimal stimulation strategy comparing monopolar and bipolar neural stimulation (Fig. 6). As clearly seen in Fig. 6b-c, thanks to this operation several additional fascicles (yellow color) can be selectively recruited compared to the monopolar stimulation (green color) both for FINE and TIME implanted in distal or proximal part of the sciatic nerve. These results were confirmed with all tested TIME and FINE regardless the number of active sites.

Overall, with the bipolar stimulation, we have observed 12.29 ± 4.7% and 8.9 ± 2.07% improvement in the number of fascicles selectively recruited in comparison to standard monopolar activity for TIME and FINE respectively (Fig. 6b,c).

**Fig. 7:**
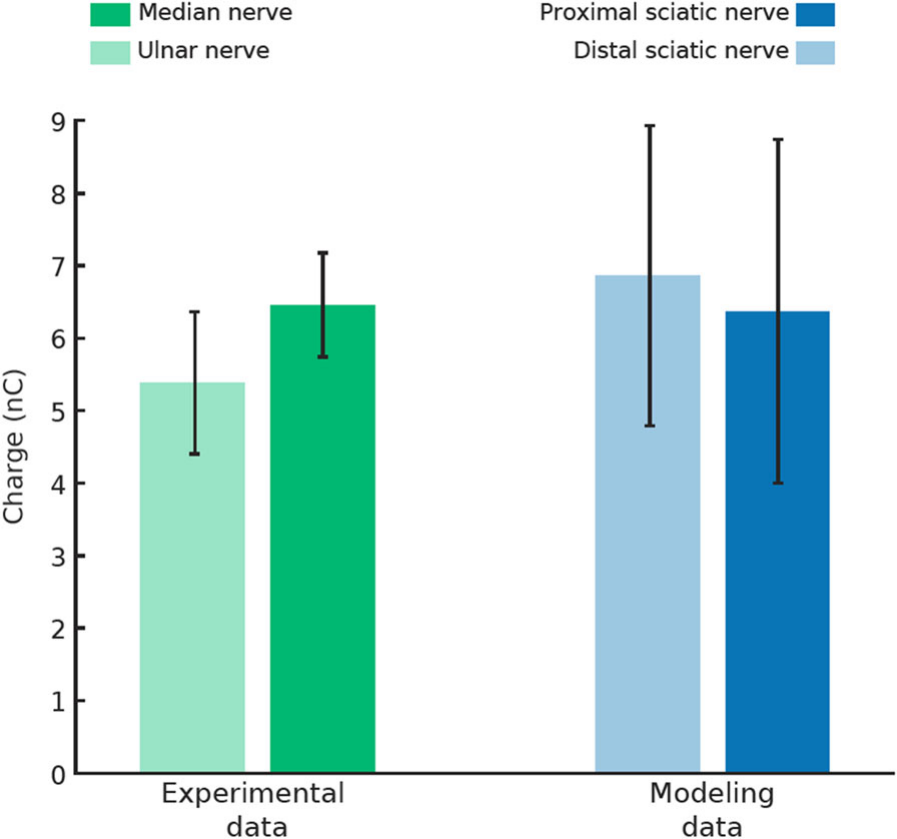
Validation of the modeling physical construction. Comparison between modeled and experimental data of minimum charges necessary to reach the perceptual threshold are shown (*p* > 0.05). Thresholds are not different (*p* > 0.05) also between different nerves both in experimental and modeling data

To benchmark our model results against available human data, we compared the thresholds values between simulated data using hybrid modelling and experimental data presented in Petrini et al., 2018 ([32]) (Fig. 7). The minimal charges necessary to selectively recruit at least 10% of the fascicle fibers ([42]) were calculated for both proximal and distal section of the sciatic nerve implanted with a TIME. They were compared for both ulnar and median nerve thresholds collected in a trans-radial amputee stimulated using implanted TIME electrodes (14 active sites). The stimulation frequency was fixed to 50 Hz ([28]). Experimental data were acquired in the first weeks of implant for all active sites (4 TIMEs × 14 active sites). No significant difference was found between experimental and modelling data (Kruskal-Wallis test with Tukey-Kramer post-hoc, *p* > 0.1), indicating a good validity of modeling results in respect to real human data. In particular, experimental thresholds were 5.39 ± 0.98 nC for the median and 6.46 ± 0.72 nC for the ulnar nerve and modelling data were 6.86 ± 2.07 nC for the proximal and 6.37 ± 2.37 nC for the distal sciatic nerve section. As expected, in both experimental and simulated data the different geometrical shape of the targeted nerve did not affected the threshold values (Kruskal-Wallis test with Tukey-Kramer post-hoc, *p* > 0.05).

## Discussion

We developed a detailed computational model of the sciatic nerve for the purpose of development of the sensory neuroprosthesis for highly disabled, lower limb amputees. It holds potential to help in transferring of promising results obtained in the upper limb to the lower-limb amputees [23, 24].

The hybrid modeling is an important step in designing optimal neural interfaces, and also to perform efficient manufacturing avoiding unnecessary animal experimentation [46]. Moreover, it allows proposing the indications for the neurosurgical procedure. Developed models [42, 43, 55, 61] for the invasive stimulation of the peripheral nerves, were mainly devoted to the design and the validation of the motor fibers, and therefore muscular activation. An interesting probabilistic modeling [43] of the sciatic nerve stimulation has been proposed, but accounting only for the motor effects, with only FINE electrodes implemented. Therefore, in the overall context, present is one of the first models with an aim of sensory neuroprosthesis design.

Physically, we are exploring different sizes of fascicles, and their arrangement, without any assumption about their specific function or placement in the specific patient. Also we are emulating a range of different fibers populations in each of these, since it is unknown how are single fibers grouped within them. We are accounting for a very small nerve specimen, and a huge one, which can correspond to the proximal and distal section, or to the huge and small individual [47, 48]. The number of fascicles accounted in the model is in the range of the sciatic nerve specimens from literature [47, 48]. Therefore, the hybrid modeling is taking into account many different physically and anatomically plausible inputs in order to obtain the “average” statistically important results, which are then generalizable.

Due to the present limitations in imaging techniques, and computational power presently available, in no way we are trying to implement the “patient-specific” devices (not to exclude in the future when imaging gets more selective, and computers more powerful), but rather to propose the indications for general device use and their design.

The MRG model adopted in this study has been originally developed only for motor fiber modeling but could be adapted to sensory Aβ fibers as well. We implemented the realistic population diameters found in the sensory fibers connected to the foot receptors [19, 20].

We performed the validation of our model results with respect to the human experimental studies, in which TIMEs were implanted in the upper limb amputees. Physically and as indicated by our results, the charge values related to the fiber thresholds (indicating that a limited subset of fibers was elicited) should be similar also in different nerve geometries, since they are calculated at the intra-fascicular level.

Model limitations include the need for the better representation of the most external layer of the nerve (defined as paraneurium [64]), which is typically not accounted [42, 43, 55, 61] for, and could play a very important role, especially when extraneural (FINE) stimulation is performed. An emulation of the nerve compression with FINE (as for the femoral nerve in Schiefer et al., 2008 [55]) could be implemented in the future, as currently implemented model closer resembles a FINE without compression (yet clinically relevant since, similar to the cuff electrodes used in the sensory feedback restoration with trans-tibial amputees [53]). A compression model including mechanical characteristics of the sciatic nerve and its fascicles would allow to properly model a deformation caused by FINE, once this experimental data becomes available.

Also, the validation with FINE experimental data should be performed, which here was impossible since we did not have access to that data. Moreover, instead of comparing thresholds found in upper-limb amputees (eg. median and ulnar nerve stimulation), the data from lower limb amputees (eg. sciatic nerve stimulation) will be compared to our modelling results for a better validation. We have used two different anatomies to emulate the anatomical variability, but in future the use of more histological sections could potentially give even higher precision of the model.

It is of the paramount importance to emphasize that, when dealing with models, they can be used properly only when addressing a clearly defined issue, and it cannot be intended to explain all the aspects of such complex system as neural system stimulation in every its aspect. Indeed, here we give indications about correct dimensioning, number of implants, and novel stimulation policies for the studied two types of electrodes in the specific sciatic nerves, which could potentially drive the development of a new generation of neuroprosthetic devices.

Definition of the “optimal” neural interface takes into account the high selectivity as the quality measure, which would be translated in reality to the discrete areas and a single type of sensation reported by amputees. They also have to account with i) low invasiveness; ii) high stability: mechanical and functional and iii) low activation thresholds, which would indicate a smaller tissue damage and a longer battery life.

We believe that with the future development of the neuro-technologies, the sophisticated and widespread neuroprosthetic devices will go towards the personalized [65] modeling-based approach. Indeed, we could think of having the patient-specific neural interface with a tuned protocol of use in the near future. Additionally, developing valid computational models not only would be a cost-efficient option for neural interfaces design, but also would reduce the number of unnecessary animal experiments (still fundamental in current neuroprosthesis development).

From the neurophysiological viewpoint, the postural reflexes are generated at the spinal level [66]. On the higher level, the information conveyed from the lower limbs into the spinal cord, and then further to the Gracile Nucleus and higher structures. It is reasonable to believe that if restore physiologically plausible sensory feedback from the missing foot and leg, these could be properly interpreted and integrated by the higher structures, achieving the correction of the incorrect sensorimotor integration occurring in lower-limb amputees. Therefore, it is of a paramount importance to design an optimal peripheral encoding for the success of such prosthetic device.

## Conclusions

We developed and validated an anatomically realistic, computational model of the sensory stimulation for the sciatic nerve. It suggests the optimal geometry of interfaces to be used in human subjects with lower limb amputation, their surgical placement and beneficial bipolar policy of stimulation. The results suggest that a highly selective stimulation of fascicles of the human sciatic nerve, which innervates the majority of sensations from the foot and lower leg, can be obtained by TIMEs and FINEs, when using very penalizing selectivity indexes. A 20-active site TIME is able to selectively activate the largest number of fascicles, in both anatomies studied. FINEs of 16 and 20 active sites resulted in highest extraneural selectivity. Simulations indicate that optimal number of TIME implants to be surgically placed in the huge sciatic nerve is three, since with addition of more electrodes there is no functional gain. Finally, with both types of electrodes the bipolar stimulations augmented significantly the performance achieved. These results will potentially enable the clinical translation of the sensory neuroprosthetics towards the lower limb applications.

## Data Availability

Data and materials used for the production of the results of the paper available from the corresponding author upon a reasonable request.

## Abbreviations

ASs: Active sites
ENM: Electro-neuro model
ePNS: electrical Peripheral Nerve Stimulation
FEM: Finite elements method
FINEs: Flat interface nerve electrodes
MRG: McIntyre-richardson-grill
PLP: Phantom limb pain
TF: Trans-femoral
TIMEs: Transversal intraneural multichannel electrodes
